# Case Report: Endoscopic ultrasound in the diagnosis and endoscopic treatment of a delayed-presentation duodenal web in a toddler

**DOI:** 10.3389/fmed.2026.1856865

**Published:** 2026-06-05

**Authors:** Qian Huang, Rensen Jiang, Miaobing Wu, Jiexing Long, Jing Lao, Dongling Dai, Liang Liu, Bin Wang, Jianyao Wang

**Affiliations:** 1 Shenzhen University Medical School, Shenzhen University, Shenzhen, Guangdong, China; 2 Department of General Surgery, Shenzhen Children’s Hospital, Shenzhen, Guangdong, China; 3 Shenzhen Pediatrics Institute of Shantou University Medical College, Shenzhen, Guangdong, China; 4 Department of Endoscopy Center, Shenzhen Children’s Hospital, Shenzhen, Guangdong, China; 5 Shenzhen Children's Hospital, Affiliated to School of Medicine, Southern University of Science and Technology, Shenzhen, Guangdong, China

**Keywords:** case report, delayed-presentation duodenal web, duodenal obstruction, endoscopic ultrasound, endoscopic web incision, pediatric patients

## Abstract

Duodenal web is rare in children, and reports of symptomatic cases with delayed presentation are even scarcer. This paper reports a 19-month-old female toddler admitted to hospital due to recurrent vomiting. Imaging examinations suggested duodenal obstruction, with an initial consideration of superior mesenteric artery syndrome or space-occupying lesion of the duodenum. In this case, circumferential thickening of the muscular layer of the duodenal wall was detected by endoscopic ultrasound (EUS), and pathological biopsy was performed simultaneously. Neoplastic lesions were systematically excluded, and the diagnosis of delayed-presentation duodenal web was established. The toddler successfully underwent endoscopic web incision eventually, avoiding open surgery. Postoperatively, the vomiting symptom was completely relieved without any complications. EUS clearly delineates the layered structure of the duodenal wall, thereby helping to elucidate the etiology of the obstruction and rule out neoplasms. This provides a direct basis for informed clinical decision-making regarding minimally invasive treatment of atypical, delayed-presentation duodenal obstruction.

## 1 Introduction

Duodenal web is one of the important causes of congenital duodenal obstruction in children (1). Most children present with typical obstructive symptoms in the neonatal period, and very few cases present with delayed onset. Up to now, only Zimmer et al. have reported a 4-year-old boy with chronic vomiting who was suspected of having delayed-presentation duodenal web by endoscopy worldwide (2). The clinical manifestations of delayed-presentation duodenal web are atypical, often only presenting as intermittent vomiting or dyspepsia, which is easily confused with superior mesenteric artery syndrome, space-occupying lesions or inflammatory diseases. Traditional diagnostic approaches mainly rely on imaging examinations or surgical exploration, which are associated with a high risk of misdiagnosis or unnecessary exploratory laparotomy, and there is no standardized diagnosis and treatment process. This paper reports a 19-month-old toddler diagnosed with delayed-presentation duodenal web. Circumferential thickening of the muscular layer of the duodenal wall was detected by EUS, and a definite diagnosis was made by pathological biopsy. The toddler successfully underwent endoscopic web incision. To our knowledge and according to the literature published to date, this represents the first case report of a delayed-presentation duodenal web managed through EUS-assisted diagnosis and endoscopic treatment, offering a novel clinical reference for the management of similar patients.

## 2 Case description

### 2.1 Case data

A 19-month-old female toddler was admitted to hospital due to “recurrent vomiting for 6 days.” The toddler developed postprandial vomiting without obvious inducement 6 days ago, with non-projectile emesis of gastric contents, and had a healthy past medical history. Before admission, the toddler was treated for “gastroenteritis” in another hospital, with no obvious relief of symptoms and accompanied by transient fever. The toddler was conscious and alert with normal mental status, no obvious dehydration, and adequate nutritional status. Abdominal examination revealed mild distension without tenderness or rebound tenderness; no mass was palpable. Abdominal tympany was noted on percussion, and bowel sounds were active. Digital rectal examination showed an empty rectum without feces; no passage of stool or flatus occurred after withdrawal of the finger. The detailed clinical timeline of the patient is shown in Table 1.

**Table 1:** Timeline of clinical manifestations and examinations.

Date	Clinical timeline
2025-08-21	Symptom onset
2025-08-22	Seeking medical care at local hospital
2025-08-27	Transfer admission to our hospital
2025-08-27	Ultrasonography
2025-08-28	Contrast-enhanced total abdominal CT scan
2025-08-30	Upper gastrointestinal contrast study
2025-09-01	Endoscopic ultrasonography with pathological biopsy
2025-09-08	Surgical operation

### 2.2 Differential diagnosis

**Figure 1:**
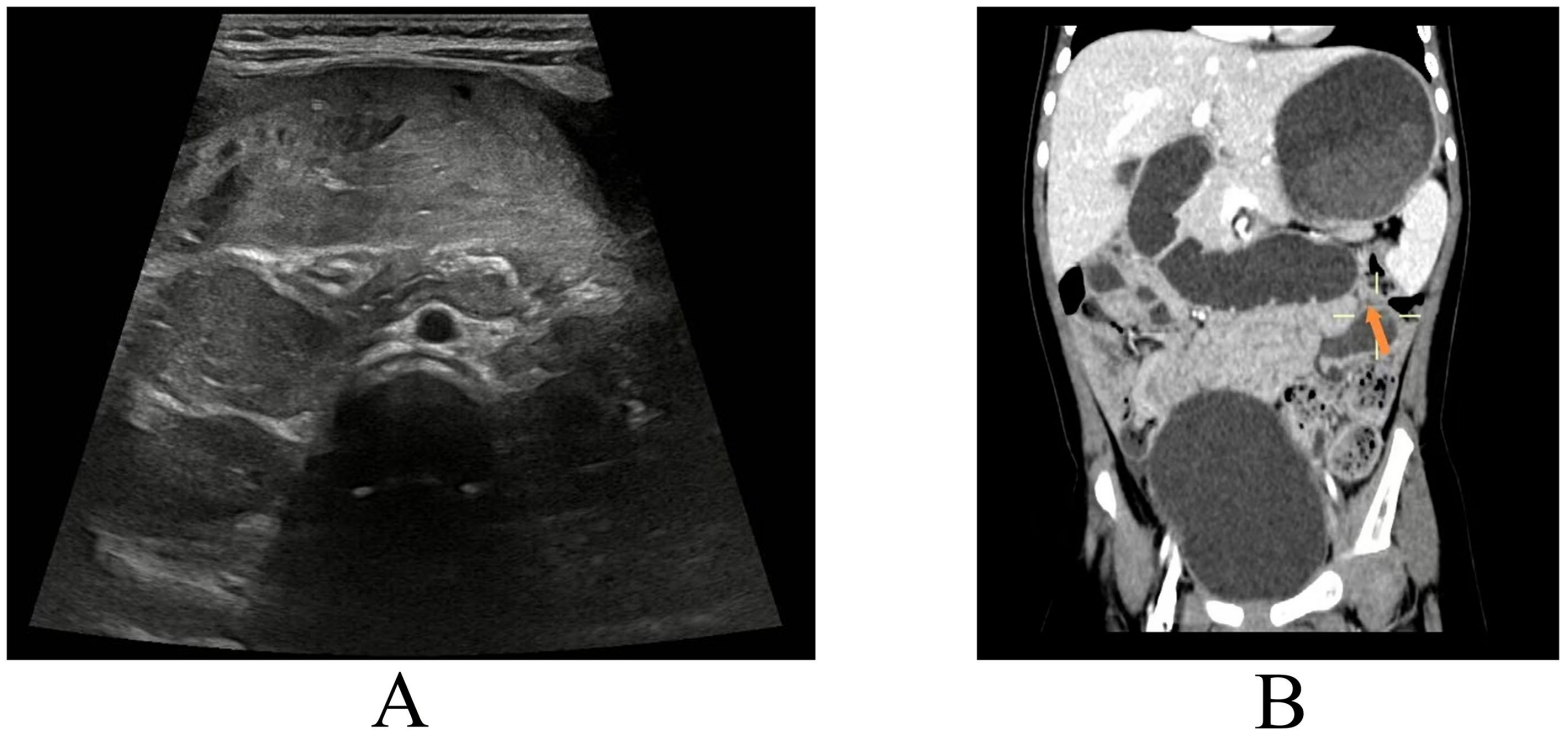
**(A)** Gastrointestinal ultrasound suggesting duodenal dilatation. **(B)** Abdominal CT suggesting high-grade incomplete mechanical intestinal obstruction, possibly caused by a foreign body or space-occupying lesion (indicated by the yellow arrow).

Gastrointestinal ultrasonography showed duodenal dilatation, and no obvious abnormalities were found in the course and diameter of the superior mesenteric artery and celiac trunk (Figure 1A). The child presented with vomiting and other related symptoms, which were similar to partial manifestations of superior mesenteric artery syndrome (SMA syndrome). Therefore, it was included in the differential diagnosis. According to the diagnostic criteria for SMA syndrome, a confirmed diagnosis requires typical clinical symptoms, characteristic imaging findings of vascular compression, and exclusion of other causes. This case lacked imaging evidence of vascular compression. Combined with subsequent endoscopic ultrasound (EUS) and pathological findings that clarified the exact cause of obstruction, SMA syndrome was excluded through exclusive differential diagnosis (3).

### 2.3 Examinations and treatment

After admission, plain and enhanced abdominal computed tomography (CT) scans were performed, which revealed an abnormal density shadow in the intestinal lumen from the distal duodenum to the jejunal transition, accompanied by obvious dilatation of the proximal duodenum and stomach, suggesting high-grade incomplete mechanical intestinal obstruction, which may be caused by a foreign body or space-occupying lesion (Figure 1B). Upper gastrointestinal barium meal examination showed difficult passage of contrast medium through the horizontal part of the duodenum, with partial passage after changing body position, suggesting local incomplete obstruction.

**Figure 2:**
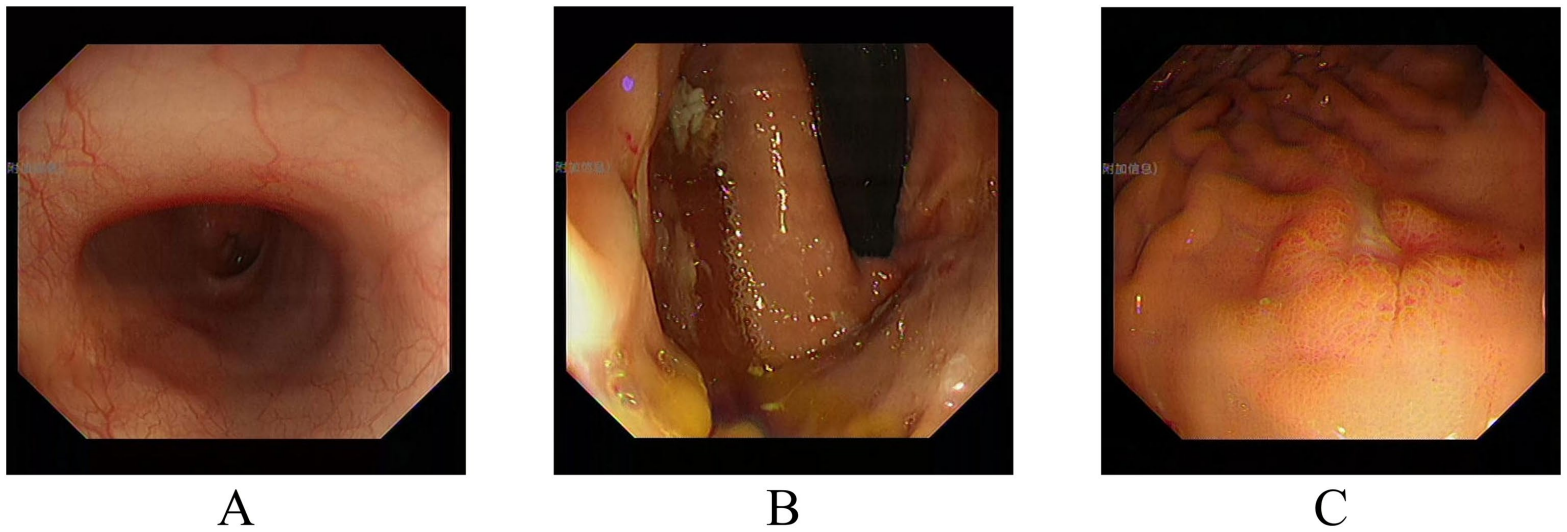
**(A)** Endoscopic image of the esophagus, showing intact esophageal mucosa with normal contraction and relaxation. **(B)** Gastric fundus and cardiac orifice with good opening and closing, no ulcers or masses seen. **(C)** An ulcer with a size of about 3 × 5 mm seen on the greater curvature of the gastric body.

**Figure 3:**
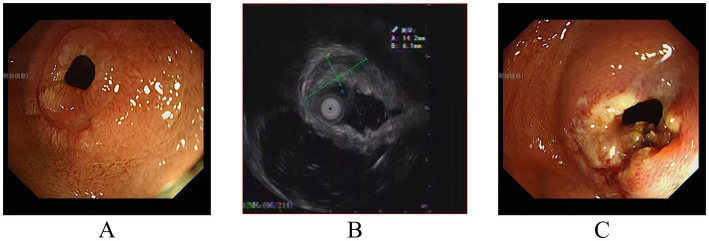
**(A)** Circumferential membranous stenosis in the horizontal part of the duodenum, with local mucosal congestion and edema and white fur covering the surface. **(B)** Thickening of the submucosa and proper muscular layer at the stenotic site with clear layers. **(C)** Unobstructed duodenal lumen after web incision.

The treatment team used EUS to examine the toddler’s duodenum. Intraoperative findings were as follows: no abnormality was found in the esophageal mucosa with good contraction and relaxation (Figure 2A); the gastric fundus and cardiac orifice had good opening and closing without ulcers or masses (Figure 2B); an ulcer with a size of about 3 × 5 mm was seen on the greater curvature of the gastric body (Figure 2C); circumferential membranous stenosis was found in the horizontal part of the duodenum near the beginning of the jejunum, with circumferential thickening of the intestinal lumen forming a narrow small hole, local mucosal congestion and edema, and white fur covering the surface (Figure 3A). EUS revealed circumferential thickening of the submucosa and muscularis propria with clear and continuous layers, without invasive growth, which was consistent with the intramural structural characteristics of duodenal web. (Figure 3B) Intraoperative pathological biopsy was conducted on the thickened lesional tissues. Specimens were collected from the mucosa of the horizontal duodenum. Two grayish-white soft tissue samples were obtained, each measuring approximately 0.1 cm × 0.1 cm × 0.1 cm. The biopsy penetrated deep into the mucosal layer, and the tissue volume was sufficient for pathological observation. Postoperative pathological biopsy and immunohistochemistry indicated chronic active inflammation without dysplasia or malignant features, ruling out the possibility of tumor.

Based on the pathological findings, duodenal web incision was further performed. The operation was carried out using an Olympus GIF-HQ290 electronic gastroscope, and multiple incisions were made on the lesional web with disposable mucosal knives produced by Jiangsu Weikang (Figure 3C). Slight oozing was observed at the incision site. Hemostasis was achieved by spraying norepinephrine, and no progressive bleeding occurred. The duodenal lumen returned to patency after the operation.

### 2.4 Prognosis and Follow-up

Postoperatively, the toddler’s vomiting symptom was completely relieved with good eating, without bleeding, perforation or other complications. Follow-up by telephone at 1 and 3 months after surgery showed that the toddler had no discomfort such as vomiting or abdominal distension when eating all kinds of food, with ideal weight gain and no abnormalities found.

## 3 Discussion

Duodenal web is an intraluminal membranous malformation caused by impaired recanalization of the intestinal lumen during the embryonic period. The onset time of its clinical manifestations depends on the size of the web orifice, and the common age of onset is the neonatal period (4). The case reported by Zimmer et al. was a 4-year-old child (2), and the onset age of the toddler in this case (19 months) was later than the typical neonatal period but earlier than 4 years old. The key common feature of these two cases is that obstructive symptoms appeared long after birth, which defines the essence of its “delayed presentation.” These two cases remind us that duodenal web should also be included in the differential diagnosis for children with unexplained chronic vomiting, especially older children.

Theoretically, duodenal web can occur in any segment of the duodenum, but it is most commonly located in the descending duodenum (second part) (4). In this case, the web was located in the horizontal segment (the third segment) of the duodenum, which is a relatively rare anatomical variation, and this also increased the difficulty of preoperative imaging localization and differential diagnosis. In view of the gastric ulcer incidentally detected during surgery in this patient, according to the findings of Mousavi SA et al., the ulcer resulted from gastric mucosal injury induced by chronic gastric content stasis and delayed gastric emptying secondary to distal duodenal obstruction, rather than bile reflux (5).

The diagnosis of duodenal web relies on imaging methods such as ultrasound. Ultrasound can sometimes detect the intraluminal duodenal web;however,it is frequently unrecognizable owing to interference by intestinal gas and contents (6). In this case, a variety of imaging methods were comprehensively used for differential diagnosis. The examination results of B-ultrasound and CT alone were inconsistent for this toddler, and both examinations had their limitations, failing to make a definite diagnosis. Therefore, EUS was specially used for intraoperative diagnosis of the toddler. Taking advantage of EUS to examine the obstructive lesion of the toddler, it was found that the submucosa and proper muscular layer were significantly thickened but with clear and continuous layers, initially ruling out neoplastic lesions and providing a basis for subsequent treatment. EUS is a new emerging technology in recent years, which has obvious advantages in the diagnosis of duodenal obstruction. It combines the advantages of endoscopy and ultrasound, and can not only examine the intraluminal condition of the intestine, but also identify neoplastic lesions in the full thickness of the intestinal wall. To the best of our knowledge, this is the first case report describing the use of EUS in the diagnosis and differential diagnosis of delayed-presentation duodenal web, which clarified the etiology, ruled out neoplastic lesions, and avoided unnecessary diagnostic laparotomy. Based on the EUS and pathological findings of this case, the patient successfully underwent endoscopic duodenal web incision. As shown in the case description, postoperative vomiting was completely resolved, and follow-up showed favorable recovery, thus successfully achieved accurate diagnosis and minimally invasive treatment of delayed-presentation duodenal web.

## 4 Conclusion

In summary, for atypical and delayed-presentation duodenal obstruction, especially when imaging examinations suggest space-occupying lesions or local stenosis, EUS-guided biopsy combined with an endoscopy-based diagnostic and therapeutic strategy enables accurate diagnosis, minimally invasive intervention, and avoidance of unnecessary surgical exploration. This case provides reference clinical experience for the diagnosis and EUS treatment of delayed-presentation duodenal web in children.

## Data Availability

The original contributions presented in the study are included in the article/supplementary material, further inquiries can be directed to the corresponding author/s.
